# Prepregnancy Obesity, Maternal Dietary Intake, and Oxidative Stress Biomarkers in the Fetomaternal Unit

**DOI:** 10.1155/2019/5070453

**Published:** 2019-06-13

**Authors:** Ana Karen Ballesteros-Guzmán, Claudia E. Carrasco-Legleu, Margarita Levario-Carrillo, Dora Virginia Chávez-Corral, Blanca Sánchez-Ramírez, Edgar Omar Mariñelarena-Carrillo, Fabiola Guerrero-Salgado, Sandra Alicia Reza-López

**Affiliations:** ^1^Facultad de Medicina y Ciencias Biomédicas, Laboratorio de Embriología, Universidad Autónoma de Chihuahua, Circuito Universitario, Campus II, Chihuahua, Chihuahua, C.P. 31109, Mexico; ^2^Facultad de Ciencias de la Cultura Física, Laboratorio de Bioquímica para la Actividad Física, Universidad Autónoma de Chihuahua, Circuito Universitario, Campus II, Chihuahua, Chihuahua, C.P. 31109, Mexico; ^3^Facultad de Ciencias Químicas, Laboratorio de Biotecnología III, Universidad Autónoma de Chihuahua, Circuito Universitario, Campus II, Chihuahua, Chihuahua, C.P. 31109, Mexico

## Abstract

**Background:**

Obesity and pregnancy increase levels of maternal oxidative stress (OS). However, little is known about the maternal, placental, and neonatal OS status.

**Objective:**

To analyze the relation between prepregnancy obesity and the expression of OS markers and antioxidant capacity in the fetomaternal unit and their association with dietary intake.

**Methods:**

This cross-sectional study included 33 women with singleton, noncomplicated pregnancies. Two groups were formed: women with prepregnancy body mass index (pBMI) within normal range (18.5-24.9 kg/m^2^, n = 18) and women with pBMI ≥ 30 kg/m^2^, suggestive of obesity (n = 15). Dietary and clinical information was obtained by questionnaire and from clinical records. Total antioxidant capacity (TAC) and malondialdehyde (MDA) concentration were measured on maternal and cord serum by colorimetric techniques, and placental expression of glutathione peroxidase 4 (GPx4) was measured by immunohistochemistry.

**Results:**

Placental GPx4 expression was lower in the group with pBMI suggestive of obesity than in the normal weight group (ß = -0.08, p = 0.03, adjusted for gestational age and magnesium intake). Concentrations of TAC and MDA in maternal and cord blood were not statistically different between groups (p>0.05). Cord MDA concentration was related to maternal MDA concentration (ß = 0.40, p < 0.01), vitamin A intake (tertile 2: ß = -0.04, p = 0.40, tertile 3: ß = 0.13, p = 0.03,* vs* tertile 1), and placental GPx4 expression (ß = -0.09, p = 0.02).

**Conclusion:**

Prepregnancy obesity is associated with a decrease in GPx4 expression in the placenta, which is related to OS in the newborn. The influence of micronutrient intake on OS biomarkers highlights the importance of nutritional assessment during pregnancy and adequate prenatal care.

## 1. Introduction 

Obesity is associated with adverse pregnancy outcomes. Women with prepregnancy obesity have increased risk of hypertensive disorders, gestational diabetes, and neonatal morbidity [1, 2]. The mechanisms underlying this association are complex. However, oxidative stress (OS) may be a factor. Oxidative stress is defined as an imbalance between the generation of free radicals and the antioxidant capacity of the organism [3]. Free radicals, such as reactive oxygen species (ROS), result from metabolic activity [4]. In excess, free radicals induce cellular damage and may impair tissue/organ function [4, 5].

The antioxidant capacity of the organism is provided by exogenous and endogenous sources. Exogenous antioxidants, such as vitamin A, C, E, selenium, zinc, manganese, and magnesium [6–9], must be obtained by dietary or supplement intake. On the other hand, endogenous antioxidants comprise a set of enzymes, such as superoxide dismutases (SOD), catalase, and glutathione peroxidases (GPx) [10]. The GPx family provides antioxidant protection by reducing hydroperoxides to water or alcohols [11]. GPx isoforms are expressed in several tissues from the gastrointestinal tract, the liver, and the placenta [11, 12]. In particular, the GPx4 isoform plays an important role in protecting membranes against OS [11].

Obesity in pregnancy influences OS status [13]. In nonpregnant women with obesity, plasmatic levels of malondialdehyde (MDA), a marker of lipid peroxidation, are increased, while antioxidant markers are decreased [14]. The rise in the amount of free fatty acids [15] and lipid peroxidation, as well as the dysregulation of adipocytokines observed in humans with obesity, have been related to elevated ROS generation [5], leading to OS. Similarly, during pregnancy, plasmatic concentration of prooxidants increases whereas enzymatic antioxidants decrease [13, 16], possibly as a result of gestational metabolic and oxygen demands. In addition, the placenta is a potential source of OS for the mother and the developing fetus, because of its high metabolic activity [17].

Obesity during pregnancy may lead to an imbalance in maternal and fetal prooxidant/antioxidant status. Some studies have shown that women with obesity have increased prooxidant levels and a decreased antioxidant capacity than women with adequate weight [13]. In addition, obesity is associated with greater lipid accumulation in the placenta [18]. Furthermore, ROS generation in placentas increases according to maternal adiposity [19]. However, the information regarding the role of maternal obesity and dietary intake on OS and antioxidant capacity of the mother, the placenta, and the newborn is scarce. Therefore, the objective of this study was to analyze the relation between prepregnancy obesity and the expression of OS markers and antioxidant capacity in the maternal-fetal unit and their association with dietary intake.

## 2. Material and Methods

### 2.1. Study Design and Subjects 

In this cross-sectional study, we included 33 pregnant women who attended a local hospital for obstetrical attention. They were invited to participate and signed a written informed consent form. Women were between 20 and 40 years of age, with full-term singleton noncomplicated pregnancies. Women with comorbidities were excluded. Information on sociodemographic, nutritional, and clinical variables was obtained by questionnaires and from clinical records. Prepregnancy BMI (pBMI) was calculated from self-reported height and weight. Two groups were formed according to pBMI: normal weight group including women whose pBMI was between 18.5 and 24.9 kg/m^2^, and a group of women (n = 15) with pBMI ≥ 30 kg/m^2^, suggestive of prepregnancy obesity. They were 23 (22-27) and 26 (25-29, median and interquartile range) years old; 56% and 40% of deliveries were by partum (p = 0.37) at gestational age (38 ± 2 and 39 ± 2 weeks, p = 0.37), in the groups with normal weight or obesity, respectively. Three women were primiparous; all of them are in the group of normal weight. The study protocol was approved by the institutional ethics board (protocol registration number CEI-A-112/14, date 06/26/2014).

### 2.2. Serum Analysis

We collected maternal and umbilical cord (UC) blood samples. Samples were centrifuged to obtain serum and stored at -70°C until analysis. MDA concentration was analyzed with a modification of the Buege & Aust technique [20], using 1,3,3–tetramethoxypropane, as standard reference, which was obtained from Sigma-Aldrich (catalogue number 108383, Sigma-Aldrich, USA). Absorbance measures were performed in a UV spectrophotometer at a 535 nm wavelength. Concentration was obtained dividing the sample absorbance by the molar extinction coefficient (*ε*_535_ = 1.56 x 10^5^ M^−1^ cm^−1^). Serum total antioxidant capacity (TAC) was measured using a commercial kit (catalogue number MAK187, Sigma-Aldrich, USA). The absorbance was measured at 550 nm with a Biotek ELx800 absorbance reader (Vermont, USA). A Trolox standard curve was used as reference to calculate sample TAC concentration. The obtained values were adjusted for the sample total protein concentration, which was quantified with the Pierce bicinchoninic acid protein assay (catalogue number 23227, Thermo Fisher, USA). All analyses were performed in duplicate and a mean value was obtained. The intra-assay coefficient of variation was 5.2% for MDA and 5.8% for TAC analyses.

### 2.3. Placenta Analysis

Placentas were collected and analyzed as previously described [21]. Briefly, immediately after delivery, samples were washed with saline water to eliminate the blood clot. An approximately 2 cm^2^ sample was taken from a lesion-free area of the central region and fixed in 10% formaldehyde. Samples were imbedded in paraffin, cut into 4 *μ*m section slices, and mounted onto microscope silanized slides. Immunohistochemical analysis was performed using a commercial kit (Histostain-Plus kit, Thermo Fisher laboratories, catalogue number 859673). Samples were dewaxed and hydrated by immersing the slides in xylene, descending concentrations of ethanol, and finally in phosphate buffered saline (PBS). Then the quenching solution was applied for 15 minutes and rinsed. We applied the blocking solution (15 minutes) and incubated the sample overnight with the primary anti-GPx4 polyclonal antibody (catalogue number 10005258, Cayman Chemical, USA) in a 1:100 dilution. The samples were rinsed and incubated with the secondary antibody for 30 minutes, rinsed, and incubated with streptavidin for 15 minutes. After rinsing with PBS, the samples were stained with a fresh solution of 3,3'-diaminobenzidine-chromogen (DAB) and DAB buffer solution, rinsed with distilled water and PBS, and then dehydrated in ascending concentrations of ethanol and xylene, before resin and coverslips were mounted. A negative control was included by omitting the primary antibody. We took 6 microphotographs at 40x magnification using an Axioskop 2 Plus (Axioskop 2 Plus, Carl Zeiss Jena GmbH, Jena, Germany), equipped with camera, and the AxioVision software (AxioVision LE, v.4.6.2.0, Carl Zeiss MicroImaging GmbH, Germany). These microphotographs were converted to gray scale to measure the expression signal in the microvillous membrane [12]. Optical density was measured in non-counterstained samples using the software Image-Pro Plus, v. 4.1. (Media Cibernetics, Silver Spring, MD, USA) and a microscope BX41 Olympus equipped with a camera DP72. All measures were performed by a single observer, who was previously trained and standardized (with an interobserver and intraobserver correlation coefficient of 0.84 and 0.94, respectively) and blinded to the maternal pBMI group. Counterstaining was performed with hematoxylin in selected samples to show structure and protein localization.

### 2.4. Nutrimental Analysis

The average daily macro and micronutrient intake during pregnancy were estimated from a food frequency questionnaire, using a software for nutrition analysis (Nutrimind©, version 2.0) and food composition databases [22]. The values obtained for macro and micronutrients are expressed per 100 kcal of the total caloric value of the diet and as tertiles of intake.

### 2.5. Statistical Analysis

After exploratory and descriptive analysis, we compared the pBMI groups by t-test for independent samples, Wilcoxon rank sum test for variables with nonnormal distribution, or chi-square test for nominal variables. Linear regression models were used to assess the relation between each marker of OS and antioxidant capacity, with maternal characteristics, and nutrient intake. Optical density measures of GPx4 were transformed to logarithmic scale to reach normality. Multiple linear regression models were used to adjust for potential confounders (gestational age, dietary intake, and maternal characteristics) and to identify the main predictors of OS and antioxidant biomarkers. A p-value < 0.05 was considered statistically significant. All tests were performed using the Stata, v. 11.0 (StataCorp, College Station, TX, USA).

## 3. Results

**Figure 1:**
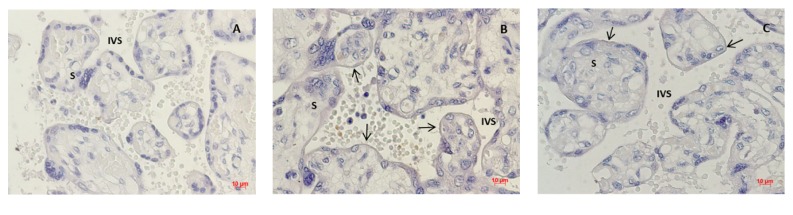
Placental Glutathione peroxidase 4 (GPx4) expression. (A) Negative control. (B) Prepregnancy BMI indicative of normal weight. (C) Prepregnancy BMI indicative of obesity. Staining is lower on those women with BMI indicative of obesity. → Indicates GPx4 expression areas in the microvillous membrane. IVS: intervillous space. S: stroma. 40x magnification.

**Figure 2:**
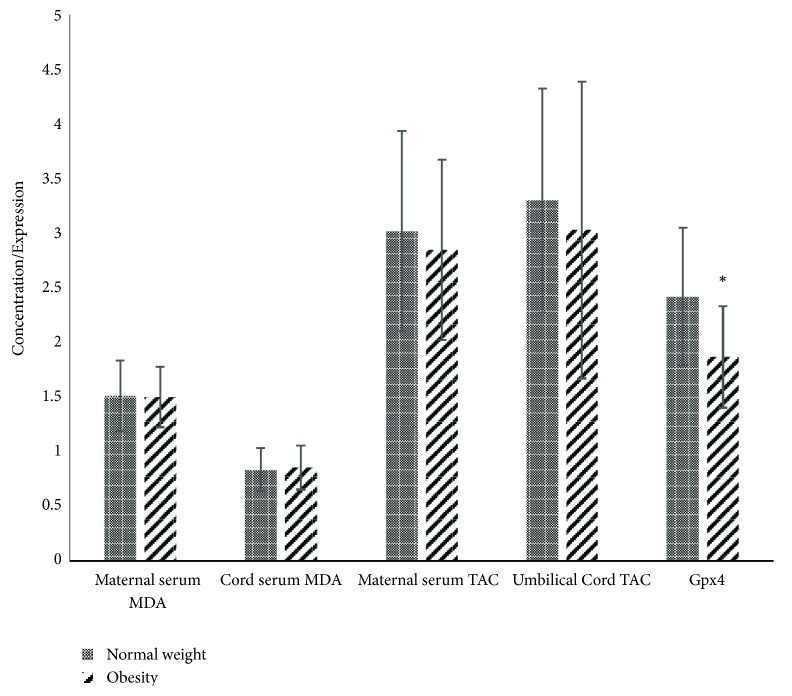
*Oxidative stress and antioxidant capacity markers in the fetomaternal unit*. GPx4 expression was lower in placenta of the group with BMI indicative of obesity (p < 0.05). Data is presented as mean ± standard deviation. MDA: malondialdehyde (*µ*mol/L). TAC: total antioxidant capacity (nmol/*µ*l/*µ*g protein). GPx4: glutathione peroxidase 4 (log of arbitrary units of optical density). *∗* p< 0.05. The concentration of MDA in cord serum in both groups was lower than in maternal serum (p < 0.01).

Estimated daily nutrient intake is shown in Table 1. There was not significant difference between pBMI groups. The expression of GPx4 was primarily located in the microvillous membrane of placentas, and lower signal was observed in placentas from the group with pBMI suggestive of obesity than in the group with normal weight (Figure 1). As shown in Figure 2, placental GPx4 expression was lower in the group pBMI suggestive of obesity than in the group with normal pBMI, but no significant difference was observed in serum markers of OS. The concentration of MDA in UC in both groups was lower than that found in maternal serum (p < 0.01). Maternal and UC serum concentration of TAC were not significantly different (p = 0.20).

Table 2 shows the bivariate relation between OS markers and selected variables, regardless of pBMI. Gestational age and vitamin C intake were positively associated with MDA concentration in maternal serum (p < 0.05). Cord MDA concentration was significantly related to maternal MDA, gestational age, and intakes of vitamin A and vitamin C (p < 0.05). Maternal TAC was inversely associated with primiparity (ß = -1.22, p = 0.02). The report of passive smoking was associated with low concentration of TAC in UC.

The multivariate analysis showed that the main predictors of MDA concentration in UC serum (R^2^ = 0.65) were maternal MDA concentration (ß = 0.40, p < 0.01), dietary intake of vitamin A (tertile 2: ß = -0.04, p = 0.40, tertile 3: ß = 0.13, p = 0.03, compared to tertile 1 of intake), and GPx4 expression in placenta (ß = -0.09, p = 0.02).

The TAC in UC serum was inversely related to maternal dietary intake of iron (tertile 2: *β* = -1.0, p = 0.03, tertile 3: *β* = - 0.74, p = 0.12, compared with tertile 1 of intake) and to the report of passive smoking (ß = - 1.0, p = 0.04, R^2^ = 0.24).

In the multivariate analysis, the expression of GPx4 (log-transformed) remained lower in placentas of the group of women with prepregnancy obesity (ß = -0.08, p = 0.03) after adjusting for gestational age (ß = -0.08, p = 0.001) and magnesium average intake (ß = 0.03, p = 0.04) than in the group of normal weight (R^2^ = 0.38).

## 4. Discussion

Prepregnancy obesity was associated with a decreased placental expression of GPx4. This association was significant after adjusting for gestational age and dietary intake. Irrespective of pBMI group, neonatal MDA concentrations were related to maternal high vitamin A intake and MDA concentrations and to low GPx4 expression in placenta. The report of passive smoking and high iron intake were associated with low TAC concentrations in cord serum.

Consistent with our results, previous studies have shown that GPx4 is expressed in several locations, including the syncytiotrophoblast, in the placenta [12]. The expression of this enzyme has been found to be decreased in patients with preeclampsia [12, 23]. In this study, we report a decrease in GPx4 associated with prepregnancy obesity in noncomplicated pregnancies. To our knowledge, this is the first report of the association between maternal obesity and the placental expression of GPx4 in the microvillous membrane. Our results are in line with reports in nonpregnant women, in which an inverse correlation (r = -0.42) was observed between BMI and GPx activity in erythrocytes [14]. The expression of GPx4 in the placenta in our study was unrelated to maternal MDA and TAC. Analyzing enzymatic activity and its association with maternal biomarkers could provide information about the role of maternal environment in antioxidant enzymes. Malti et al. (2014) examined OS markers in maternal, cord blood, and placenta samples. They found an increase in OS markers, and high activity of catalase and SOD in placentas from women with obesity [13]. This suggests that maternal obesity influences oxidant/antioxidant balance in the placenta.

Several vitamins and oligoelements contribute to antioxidant capacity; therefore, we examined the influence of maternal dietary intake on OS. Women in the lowest tertile of the estimated average intake of magnesium showed decreased expression of placental GPx4. Consistently, in pregnant women with preeclampsia, a correlation has been found between GPx activity and plasmatic concentration of magnesium [24]. Evidence from animal studies shows that the activity of GPx is lower in the liver of rats with a magnesium-deficient diet [25]. However, the mechanisms explaining magnesium role in OS and antioxidant capacity are still unclear.

The concentration of MDA in maternal serum in our study was increased in more advanced gestational age and in women with the highest iron intake, irrespective of pBMI. In contrast, other studies have reported higher values of MDA in women with prepregnancy obesity and/or in their newborns than in normal weight groups [13, 26]. The relation of MDA concentrations with gestational age has been reported by some authors, in agreement with our results [27]. However, other studies have not found a significant change by gestational age in healthy women [28]. Differences across studies may be explained by characteristics of the studied population, such as their BMI. Regarding dietary factors associated with OS, studies in animal models indicate that both low and high iron intakes increase OS, which could be attributed to its role in the production of some free radicals [29, 30]. Since endogenous and exogenous antioxidants influence OS, we included antioxidant vitamin intake on multivariate analyses, but did not show a significant association with MDA.

Taken together, our results provide evidence of the influence of maternal OS on fetal OS status and of the protective role of the placenta. The concentrations of MDA in UC were increased with increased concentrations of maternal MDA and decreased expression of GPx4 in the placenta. The inverse relation between MDA in UC and placental expression of GPx4 is consistent with the antioxidant role of this enzyme, which is responsible for the reduction of some free radicals and lipoperoxidation products to their inactive forms. The isoforms of GPx are expressed in the stroma and syncytiotrophoblast of the placental villi [12]. In this study, the measurement of the expression of this enzyme was made in the syncytiotrophoblast, which is in contact with maternal blood, suggesting that it may protect the fetus from maternal OS. Furthermore, the relation between MDA concentration in UC and maternal MDA, as well as with the expression of GPx4, suggests that the placenta plays an essential role in the regulation of OS from the maternal environment. Determining enzymatic activity could provide further information. However, it would include the activity of the placental GPx4 (i.e., syncytiotrophoblast), but also of other cell types, such as erythrocytes, that can be found in placental sample homogenates. An advantage of immunohistochemistry analysis is that it allows identifying more precisely the protein localization within the tissue. Given the complexity of the antioxidant system, further research is necessary to fully characterize the role of its components in the placenta and their influence in maternal, placental, and neonatal tissues.

A decreased expression of GPx4 in placentas of women with prepregnancy obesity could result in greater exposure of the product to OS. Oxidative stress has been related to the presence of congenital malformation [31], and, in the perinatal period, it has been suggested that OS may play a role in pathologies of the newborn [32]. Further studies are needed in order to assess the impact of OS on perinatal morbidity.

The results of this study provide information about the relation between prepregnancy obesity and OS markers and antioxidant capacity in the maternal-fetal unit. Additionally, we identified dietary intake variables and maternal characteristics associated with OS biomarkers and antioxidant capacity. The relation between variables was adjusted for potential confounders and independent predictors. The personnel involved in data collection and analysis were standardized in each of the techniques used, which would reduce potential measurement errors. However, our study has several limitations: (1) Prepregnancy weight and height were self-reported. (2) We estimated the average daily intake of nutrients from food frequency questionnaires, which rely on the subject memory and portion estimation. This method may be inaccurate for measuring individual dietary intake; however, it allows classifying high and low intakes. (3) The sample size was limited for the evaluation of some variables, such as the role of parity; nevertheless, it allowed establishing the main comparisons of interest.

## 5. Conclusions

In conclusion, prepregnancy obesity is associated with a decrease in GPx4 expression in the placenta, which is related to OS in the newborn. The influence of micronutrient intake on OS biomarkers highlights the importance of nutritional assessment during pregnancy. Due to the large number of processes involving OS and prepregnancy obesity, the results of this work provide information about the importance of preventive and therapeutic measures during prenatal and neonatal care.

## Figures and Tables

**Table 1:** Estimated daily nutrient intake during pregnancy, according to prepregnancy BMI.

Variable	Normal weight	Obesity	p
	(pBMI 18.5-24.9 kg/m^2^)	(pBMI ≥30 kg/m^2^)	
	n= 18	n= 15	
	Mean ± SD	Mean ± SD	
Energy (kcal)	2620 ± 794	2382 ± 471	0.31
Proteins (g/100 kcal)	4.4 ± 0.6	4.1 ± 0.6	0.19
Fats (g/100 kcal)	3.3 ± 0.7	2.8 ± 0.7	0.05
Magnesium (mg/100 kcal)	17.5 ± 3.1	18.9 ± 3.1	0.20
Zinc (mg/100 kcal)	0.5 ± 0.1	0.5 ± 0.1	0.53
Iron (mg/100 kcal)	1.5 ± 0.3	1.4 ± 0.4	0.60
Vitamin A (*μ*g/100 kcal)	49.0 ± 16.2	53.3 ± 14.9	0.44
Vitamin C (mg/100 kcal)	9.9 ± 4.7	10.2 ± 4.7	0.87
Vitamin E (mg/100 kcal)	0.5 ± 0.4	0.3 ± 0.3	0.23

pBMI: prepregnancy body mass index. SD: standard deviation.

**Table 2:** Bivariate relation between oxidative stress markers and characteristics of the studied population.

Variable	Maternal serum MDA (*µ*mol/L) *β* (95% CI)	Cord serum MDA (*µ*mol/L) *β* (95% CI)	Maternal serum TAC (nmol/*µ*l/*µ*g protein) *β* (95% CI)	Cord serum TAC (nmol/*µ*l/*µ*g protein) *β* (95% CI)	GPx4 (Log A.U.O.D.) *β* (95% CI)
Pre-pregnancy BMI (kg/m^2^)	-0.002	0.0009	-0.01	-0.004	-0.01
	(-0.02, 0.02)	(-0.01, 0.01)	(-0.06, 0.04)	(-0.07, 0.06)	(-0.03, 0.001)
Gestational age (weeks)	0.08*∗*	0.04*∗*	-0.01	-0.15	-0.07*∗*
	(0.02, 0.13)	(0.01, 0.08)	(-0.20, 0.18)	(-0.40, 0.10)	(-0.11, -0.03)
Type of delivery (partum vs c-section)	-0.09	-0.04	-0.20	0.22	-0.10
	(-0.31, 0.13)	(-0.18, 0.10)	(-0.86, 0.47)	(-0.69, 1.13)	(-0.27, 0.08)
Passive smoking	0.10	0.05	-0.09	-1.10*∗*	-0.07
	(-0.18, 0.39)	(-0.12, .23)	(-0.87, 0.70)	(-2.07, -0.13)	(-0.28, 0.15)
Energy (per 1000 kcal)	0.05	-0.01	0.04	-0.05	0.04
	(-0.11, 0.22)	(-0.12, 0.10)	(-0.48, 0.56)	(-0.75, 0.65)	(-0.10, 0.17)
Proteins (g/100 kcal)	0.11	0.01	0.19	-0.05	0.03
	(-0.07, 0.30)	(-0.11, 0.13)	(-0.35, 0.73)	(-0.78, 0.69)	(-0.12, 0.18)
Fats (g/100 kcal)	-0.04	-0.01	0.02	0.06	0.02
	(-0.19, 0.11)	(-0.11, 0.09)	(-0.45, 0.49)	(-0.55, 0.66)	(-0.15, 0.11)
Iron (mg/100 kcal)	0.31	0.21	0.38	-0.77	0.02
	(-0.01, 0.64)	(0.00, 0.42)	(-0.63, 1.40)	(-2.15, 0.62)	(-0.27, 0.30)
Magnesium (mg/100 kcal)	-0.0003	-0.01	-0.04	-0.05	0.005
	(-0.04, 0.04)	(-0.03, 0.01)	(-0.16, 0.08)	(-0.20, 0.10)	(-0.02, 0.03)
Zinc (mg/100 kcal)	0.80	-0.03	2.33	0.15	-0.61
	(-0.21, 1.80)	(-0.70, 0.63)	(-0.76, 5.42)	(-3.88, 4.17)	(-1.45, 0.23)
Vitamin A (*µ*g/100 kcal)	0.0005	0.01*∗*	-0.01	0.005	-0.002
	(-0.007, 0.008)	(0.001, 0.01)	(-0.03, 0.009)	(-0.02, 0.03)	(-0.008, 0.004)
Vitamin C (mg/100 kcal)	0.02*∗*	0.02*∗*	0.01	0.01	0.003
	(0.001, 0.05)	(0.01, 0.03)	(-0.70, 0.1)	(-0.10, 0.11)	(-0.02, 0.02)
Vitamin E (mg/100 kcal)	-0.08	-0.08	-0.10	0.56	0.18
	(-0.40, 0.24)	(-0.34, 0.17)	(-1.10, 0.88)	(-1.30, 2.41)	(-0.07, 0.43)
Maternal TAC (nmol/*µ*l/*µ*g protein)	0.05	0.04	-	0.32	-0.01
	(-0.08, 0.17)	(-0.05, 0.012)		(-0.21, 0.86)	(-0.13, 0.10)
Maternal MDA (*µ*M)	-	0.44	0.45	-0.93	-0.16
		(0.26, 0.61)	(-0.81, 1.71)	(-2.52, 0.68)	(-0.46, 0.14)
GPx4 (log UADO)	-	-0.38*∗*	-	-0.20	-
		(-0.66, -0.11)		(-1.00, 0.60)	

MDA: malondialdehyde; TAC: total antioxidant capacity (adjusted for serum proteins). GPx4: glutathione peroxidase 4, logarithm of arbitrary units of optical density.

*∗*p<0.05.

## Data Availability

The data used to support the findings of this study have not been made available to respect participants' confidentiality.
